# Clinical analysis of ten cases of HIV infection combined with acute leukemia

**DOI:** 10.1515/med-2024-1081

**Published:** 2025-02-24

**Authors:** Shanshan Fan, Chuan Qian, Pengfei Tao, Qiwen Zhou, Sen Lin, Konglong Li, Xi Wang, Haiyan Min

**Affiliations:** Second Department of Infection Disease, Yunnan Provincial Infectious Disease Hospital, Kunming, Yunnan 650301, China

**Keywords:** HIV, acute leukemia, antiviral therapy, chemotherapy, regression, prognosis

## Abstract

**Objective:**

To summarize the clinical characteristics, diagnosis, and treatment experience of human immunodeficiency virus (HIV) infection combined with acute leukemia.

**Methods:**

Ten patients with HIV infection (eight males, two females; mean age of 40 years) were diagnosed with acute leukemia. Clinical features, diagnosis, treatment, and outcomes of these patients were retrospectively analyzed.

**Results:**

Among these ten patients, eight acute myeloid leukemia cases and two acute lymphoblastic leukemia cases were L3; three cases of M3 were positive for the promyelocytic leukemia and Vitamin A acid receptor alpha (PML/RARA) fusion genes, and four cases presented multiple chromosomal structural and numerical abnormalities. CD4+ T cell counts of the ten patients ranged from 84 to 389 cells/μL with a mean of 253.5 cells/μL. Among six patients who received chemotherapy, three cases were alive, two died of sepsis secondary to myelosuppression after chemotherapy, and one was lost to follow-up. Among four patients who did not receive chemotherapy, three died, one had M3 treatment and died with cerebral hemorrhage, and one was lost to follow-up. The maximum survival time was 74 months.

**Conclusion:**

HIV combined with acute leukemia has a complex presentation and rapid progression, early diagnosis and timely initiation of standard chemotherapy along with active antiviral therapy can improve patient’s survival.

## 1 Introduction

With the use of antiretroviral therapy (ART), the infection of human immunodeficiency virus (HIV) has gradually transformed into a chronic disease. However, HIV-infected patients have a significantly increased risk of malignancy, particularly acute leukemia. In the past 5 years, many cases of HIV complicated with acute leukemia have been reported, revealing the clinical characteristics and treatment challenges [1]. Studies have shown that HIV infection not only weakens the immune system but may also increase the risk of acute leukemia by affecting hematopoietic stem cells and the bone marrow microenvironment. In addition, HIV-infected patients need to be especially aware of drug–drug interactions and overlapping side effects when receiving chemotherapy and ART. Although several international clinical trials have been carried out to explore new therapies including gene editing technology and targeted therapy, there is still a lack of unified guidance for the treatment of HIV complicated with acute leukemia [2]. Domestic researchers are also actively exploring treatment strategies suitable for Chinese patients, but large-scale clinical studies and in-depth mechanism discussions are still insufficient.

This study retrospectively analyzed the clinical data of ten patients with HIV complicated with acute leukemia diagnosed in Yunnan Infectious Disease Hospital from 2016 to 2021, aiming to provide an in-depth understanding of the clinical characteristics, diagnosis, and treatment experience of these patients, and to provide reference for future research directions and treatment strategies.

## 2 Methods

### 2.1 Objects

The data of ten patients with HIV infection combined with acute leukemia diagnosed in Yunnan Infectious Disease Hospital from February 2016 to June 2021 were collected. The diagnosis of HIV infection was based on the HIV-1 antibody confirmation test. The diagnosis and typing of acute leukemia were based on the French-American-British diagnostic criteria and the 2016 World Health Organization classification criteria for hematopoietic and lymphoid tissue tumors.

### 2.2 Diagnosis

The general condition, clinical manifestations, past medical history, diagnosis, treatment process, and outcome of ten patients were reviewed and collected. Laboratory tests included blood routine, liver and kidney function, lactate dehydrogenase, ferritin, and β2 microglobulin. The tests related to the evaluation of HIV infection included anti-HIV-antibody (Ab), HIV-RNA quantification, and peripheral blood CD4+ T lymphocyte count. Diagnosis of acute leukemia was based on bone marrow cytomorphology, bone marrow biopsy, immunohistochemistry, immunophenotyping, and gene and chromosome karyotype analysis. The risk levels of patients were determined for the consideration of the treatment (Table 1).

**Table 1:** Risk levels of patients to determine the treatment

Case	Hazard stratification
1	Multiple chromosomal abnormalities and subtetraploidy, high risk
2	Low risk
3	Moderate risk
4	High risk
5	Low risk
6	Low risk
7	Low risk
8	Moderate risk
9	Low risk
10	Chromosomal abnormalities (-7, del(5)(q13q31)), high risk

### 2.3 Treatment

The treatment plan of acute leukemia followed the “China Guidelines for the Diagnosis and Treatment of Acute Lymphoblastic Leukemia in Adults (2021 Edition)” [3] and the “China Diagnostic and Treatment Guidelines for Adults with Acute Myeloid Leukemia (Non-Acute Premature Myeloid Leukemia) (2021 Edition)” [4]. Evaluation of therapeutic efficacy included the complete remission (CR) defined as (1) disappearance of symptoms, no circulating primitive cells or extramedullary disease; (2) active hematopoiesis in all three lineages of the bone marrow, with <5% of primitive cells; (3) absolute count of neutrophils >1.0 × 10^9^/L; and (4) PLT > 100 × 10^9^/L. Highly active antiretroviral therapy (HAART) was used for the treatment of HIV infection. The regimen was in accordance with the “China AIDS Diagnosis and Treatment Guidelines (2021 Edition)” [5]. The choice of drugs was two nucleoside reverse transcriptase inhibitors combined with one non-nucleoside reverse transcriptase inhibitor (NNRTI) or integrase Inhibitors.

### 2.4 Follow-up

The electronic medical records of Yunnan Infectious Disease Hospital and telephone follow-up were used for follow-up. Follow-up visits were performed to assess patients’ treatment effects, complications, disease regression, and clinical outcomes. Overall survival (OS) time was defined as the time from the patient’s first visit for myeloid cytomorphology-confirmed diagnosis of acute leukemia to the time of the patient’s death or the time of the last follow-up visit (Ending November 30, 2023).


**Ethical approval:** This study was approved by The Ethics Committee of Yunnan Provincial Infectious Disease Hospital. Participants have provided their written informed consent to participate in this study.

## 3 Results

### 3.1 General clinical features at onset of acute leukemia

Patients with HIV infection combined with acute leukemia (*n* = 10) were included; eight males and two females, median age at onset of acute leukemia was 40 (23–64) years old, and no family history of leukemia. At the onset of leukemia, the median peripheral white blood cell (WBC) was 2.64 (0.61–16.4) × 10^9^/L, absolute neutrophil value was 0.49 (0.12–13.8) × 10^9^/L, platelet count (PLT 66) was (12–480) × 10^9^/L, and hemoglobin was 74 (41–125) g/L. Case 4 had a WBC of 69 × 10^9^/L on admission.

### 3.2 Clinical features of acute leukemia

Bone marrow cytomorphology and immunophenotyping examination showed eight cases of acute myeloid leukemia (AML): three cases of M4 type, three cases of M3, one case of M5, and one case of M6, and two cases of acute lymphoblastic leukemia (ALL)-L3. Three cases of M3 were positive for promyelocytic leukemia and Vitamin A acid receptor alpha (PML/RARA) fusion genes, and four patients presented multiple chromosome structure and number abnormalities. Case 10 was diagnosed with nasal natural killer/T-cell lymphoma (NKTL) in October 2014. After radiotherapy, the lymphoma was in CR. Seventy-months later, the patient presented with anemia, and was diagnosed with AML M4 by combining bone marrow cytomorphology, bone marrow biopsy, bone marrow flow, and bone marrow chromosomal karyotyping. The clinical features of the patient and the results of bone marrow, gene, and chromosomal karyotyping are shown in Table 2.

**Table 2:** Bone marrow and other related clinical indicators in patients with HIV infection combined with acute leukemia

Example number	Distinguishing between the sexes	Age	Leukemia typing	Myelodysplasia	Bone marrow primitive and naive	Genomics	Chromosome karyotyping	Ferritin ng/mL	β2-microglobulin ng/mL	LDH µ/L
1	Male	64	M4	Apparently active	0.806	No mutated genes detected	Kernel: 42–43, X,−Y,add(3)(q25),add(5)(q15),−6,−7,+8,add(10(p13),der(12)*t*(12;13)(p12;q12),−13,add(14)(p11),−16,−17,add(19)(q13,3)[cp10]	2575.18	7,043	Normalcy
2	Women	40	M6	Brighten up	0.928	No mutated genes detected	Karyotype: 46, XX [22]	—	—	Normalcy
3	Male	31	ALL-L3	Hyperactive	0.96	—	—	13582.22	—	5,436
4	Male	54	M3	Apparently active	0.93	PML/RARA fusion gene positive +	—	—	—	—
5	Male	61	M4	Brighten up	0.53	—	—	483.71	—	Normalcy
6	Women	31	M3	Apparently active	0.746	PML/RARA fusion gene: bcr-1 (L-type) positive +	Karyotype: 46, XX [22]	—	—	Normalcy
7	Male	57	M5	Brighten up	0.56	—	—	211.09	—	302
8	Male	23	ALL-L3	Brighten up	0.845	No mutated genes detected	Karyotype: 46, XY, *t*(8;14) (q24.;q32) [6]/46, XY[18], heterozygous for chromosomes 8 and 14	—	6,531	1,120
9	Male	40	M3	Brighten up	—	PML/RARa gene PCR: bcr1 (L-type) positive, bcr2 (S-type) weakly positive, bcr3 (V-type) negative	Karyotype: 46, XY, *t*(15;17)(q24;q21), *t*(17;20)(q25;q11) [22]	—	1881.44	203
10	Male	38	M4	Minimize	0.23	—	Karyotype: 46, XY,−7, inv(9)(p12q13)c[2]/45, idem, del (5)(q13q31) [16]	1851.74	1766.21	98

Note: AML: acute myeloid leukemia; ALL: acute lymphoblastic leukemia; —: not detected.

### 3.3 Clinical features of HIV infection

The duration of HIV infection before the onset of acute leukemia in ten cases ranged from 0 to 100 months, of which nine patients had regular HAART prior to chemotherapy. In one case, HIV infection was detected at the same time as the diagnosis of acute leukemia, and the patient underwent HAART after the start of chemotherapy. The patient’s peripheral blood CD4+ T-lymphocyte counts, at the time of the diagnosis of acute leukemia and HIV-RNA, are shown in Table 2.

### 3.4 Treatment and regression of acute leukemia

For three patients with AML-M4, one received DA (Zoerythromycin + Cytarabine) regimen chemotherapy and was discharged from the hospital after abandoning the treatment on the third day of the first cycle of chemotherapy; one survived after completing 10 cycles of chemotherapy, and another died after abandoning the treatment after the diagnosis of acute leukemia. For three patients with AML-M3, two were treated with vitexin, arsenous acid, and the compound Huangdai tablet regimen chemotherapy; two cases reached the CR after the first cycle of chemotherapy and are currently alive; one case died of cerebral hemorrhage on the fourth day of admission; one case of AML-M6 died after completing four cycles of chemotherapy; one case of AML-M5 gave up the treatment and died after the diagnosis. For two cases of ALL-L3, one case gave up the treatment and lost the visit after the diagnosis of acute leukemia; the other case completed two cycles of VDCLP (Vincristine + Zoerythromycin + Cyclophosphamide + Mentholase + Prednisone) regimen chemotherapy. After the first cycle of chemotherapy reached CR, the leukemia recured and the patient died 73 days from the first cycle of chemotherapy. Among the ten patients receiving chemotherapy, six of them had developed fourth-degree myelosuppression and infection, of which two and ten were infected with multiple pathogens, and the myelosuppression was prolonged and difficult to correct. Ten cases were treated with chemotherapy, and the maximum blood transfusion volume of one case with chemotherapy could reach 29.5 units. No patient received hematopoietic stem cell transplantation, and the specific chemotherapy regimen and regression are shown in Table 3.

**Table 3:** Chemotherapy regimens and regression in patients with HIV-combined acute leukemia

Example number	Chemotherapy regimen	Mitigation	Co-infection after chemotherapy	Lapse to	Total survival time (months)
1	DAx1	—	Lung infection	An unauthorized visit	—
2	COAPx1	PR	*Escherichia coli* septicemia	Dead	16
	DAx1				
	DOAPx2		TB		
3	—	—	No	An unauthorized visit	—
4	—	—	No	Dead	0
5	—	—	TB	Dead	6
6	Vitamin A acid, arsenite, and compound yellow dye tablet	CR	TB	Survived	74
7	—	—	No	Dead	1
8	VDCLPx2	CR	Lung infection	Dead	2.5
9	Vitamin A acid, Arsenite and Compound Yellow Dye Tablet	CR	Lung infection	Survived	32
10	DOAPx4	CR	*Pseudomonas aeruginosa* septicemia	Survived	39
	DAx1		*Escherichia coli* septicemia		
	HOAPx1		Herpes zoster virus (med.)		
	High-dose cytarabine x3		Fungal infection		
	COAPx1		Condyloma acuminatum		
			TB		

DA: daunorubicin + cytarabine; COAP: cyclophosphamide + vincristine + cytarabine + prednisone; DOAP: daunorubicin + vincristine + cytarabine + prednisone; VDCLP: Vincristine + daunorubicin + cyclophosphamide + asparaginase + prednisone; HOAP: High-tip trialiphatic base + cytarabine + prednisone acetate tablets + vincristine; CR: complete remission; —: Not tested or not treated.

### 3.5 Other concomitant diseases

Before admission, Case 3 was with pneumonia, and the right kidney with pain; Case 5 was with pulmonary tuberculosis (TB); Case 6 was with osteoporosis; Case 7 was with peripheral facial paralysis and fungal enteritis; Case 9 was with coagulation dysfunction and hypertension; 10 cases were with nasal NKT lymphoma, and 10 cases were AML: all 4:1. The main symptoms are fever (3), bleeding (4), anemia (2), and abdominal distension (1). CD4+ T cell counts ranged from 84 to 389/μL, with a mean of 205.6/μL, a median of 158/μL, 0 (0%) < 50/μL, 50 (60%) for 50–199/μL, 4/200/μL (40%), and CD4+ T cell counts were less than 350/μL in 10 patients, and most patients were severely immunocompromised. Ten patients had a minimum duration of HIV of 0 months and a maximum of 100 months. ART before a confirmed diagnosis of acute leukemia was performed for a minimum of 0 months and up to 89 months, and three of the ten patients in this study underwent HIV-RNA quantification. All of them were less than the lower limit of detection. Nine of the ten patients were diagnosed with acute leukemia with HIV infection, nine with dotted ART number, and one patient was found to be co-infected with HIV when diagnosed with acute leukemia, and antiviral therapy was initiated at the same time as anti-leukemia therapy.

### 3.6 Clinical outcomes after ART

The regimen was tenofovir + lamivudine + lattiravid potassium tablets. The medication regimen was in line with the recommendations of the Chinese Guidelines for the Diagnosis and Treatment of AIDS (2021 Edition) [5]. A total of ten patients were diagnosed with HIV infection and acute leukemia. Among the ten patients, there were eight cases of AML and two cases of ALL. Among them, three patients with M3 (acute promyelocytic leukemia) were detected with PML/RARA fusion genes, and four patients showed multiple chromosomal structure and quantity abnormalities. CD4+ T cell counts ranged from 84 to 389 cells/µL in ten patients, with a mean of 253.5 cells/µL, suggesting that most patients were severely immunocompromised. Of the six patients who received chemotherapy, three survived, two died due to myelosuppression complicated by infection after chemotherapy, and one was lost to follow-up. Of the four patients who did not receive chemotherapy, three died, one died due to intracerebral hemorrhage after treatment for M3 leukemia, and one was lost to follow-up. The longest survival time is 74 months.

### 3.7 Clinical outcomes after HAART

In the ten patients studied, the duration of HIV infection ranged from 0 to 100 months. This suggests that patients might develop acute leukemia even within a relatively short period of time after the diagnosis of HIV infection. Nine patients had received regular HAART prior to chemotherapy, which may have an impact on the control of HIV infection and the onset of leukemia. One patient was only found to have HIV infection when he was diagnosed with acute leukemia and received HAART after chemotherapy was started. CD4+ T cell counts in all ten patients were below normal (<350/μL), suggesting that most patients had severely compromised immune systems, which might be related to the duration and severity of HIV infection. Of the six patients who received chemotherapy, three survived, which may be related to the length of time they received HAART and the control of HIV infection. However, no direct correlation has been found between the duration of HIV infection and treatment response or survival outcomes. Although the duration of HIV infection may affect a patient’s immune status and leukemia risk, there is a wide variation between individuals, and a variety of factors (e.g., genetics, environment, lifestyle, etc.) could affect the final outcome (Table 4).

**Table 4:** HIV-related assessment indicators and HAART protocol in patients with HIV-infection combined with acute leukemia

Example number	Date of diagnosis of HIV	Date of diagnosis of leukemia	HIV duration (months)	CD4+ T-lymphocyte count (individuals/µL)	HIV-RNA quantification (copies/mL)	HAART time before chemotherapy (months)	HAART
1	2014-6-13	2019-04-30	60	292	—	60	TDF + 3TC + LPV/r
2	2008-11-17	2017-03-22	100	129	0	67	TDF + 3TC + LPV/r
3	2020-06-20	2021-06-02	12	349	—	12	TDF + 3TC + EFV
4	2013-07-4	2016-02-18	31	302	—	31	TDF + 3TC + EFV
5	2013-09-07	2021-04-15	96	295	—	84	EVG/Cobi/FTC/TAF
6	2013-4-01	2017-08-29	52	149	—	52	TDF + 3TC + RAL
7	2015-08-25	2019-10-01	24	160	—	24	TDF + 3TC + EFV
8	2018-09-12	2018-09-12	0	159	—	0	TDF + 3TC + RAL
9	2018-10-24	2021-03-04	36	84	0	36	TDF + 3TC + EFV
10	2014-03-26	2021-08-19	89	137	0	89	TDF + 3TC + EFV

Note: HAART: highly active antiretroviral therapy, TDF: tenofovir; 3TC: lamivudine; LPV/r: lopinavir/ritonavir; EFV: efavirenz; EVG/Cobi/FTC/TAF: elvitegravir, cobicistat, emtricitabine, and tenofovir alafenamide tablets; RAL: raltegravir potassium; —: not tested.

### 3.8 Genetic mutations

The PML/RARA fusion gene, a characteristic hallmark of acute promyelocytic leukemia (APL), was detected in three patients with acute myeloid leukemia type M3 (AML-M3). For chromosomal aberrations, four patients exhibited multiple abnormalities in chromosomal structure and quantity. In one patient, karyotyping of bone marrow cells revealed complex chromosomal aberrations, including at least two chromosomal abnormalities, sometimes as many as six, and the presence of subtetraploidy. Patients with HIV-infected acute leukemia may have more complex chromosomal aberrations, such as multiple chromosomal abnormalities and subtetraploidy observed in a single patient. In addition, one patient mentioned in the article (the 10th patient) developed AML-M4 after 70 months of NKTL treatment, and his bone marrow karyotype showed a monosomy 7 deletion, a partial deletion of the long arm of chromosome 5, and an inversion of chromosome 9, which were associated with poor prognosis. AML-M4 patients: Among AML-M4 patients, one survived chemotherapy, while the other abandoned treatment and died after being diagnosed with acute leukemia. ALL-L3 patients: among the two ALL-L3 patients, one gave up treatment and was lost to follow-up after diagnosis of acute leukemia. Another patient completed two cycles of VDCLP chemotherapy and achieved CR after the first cycle, but leukemia relapsed and died after 73 days.

## 4 Discussion

In the present study, we have focused on AIDS cases infected with HIV combined with acute leukemia. The patients have shown complex presentation and rapid progression. Therefore, early diagnosis and initiation of standard chemotherapy along with active antiviral therapy is needed to improve the patient’s survival. The treatment of HIV infection is gradually shifting to a chronic disease management model. China is constantly raising the threshold for HIV management, as evidenced by the shift from three 90% to three 95% (95% of people living with HIV can be diagnosed, 95% of diagnosed patients are with ART, and 95% of patients with ART have viral suppression in their bodies) [5]. The interaction between ART drugs and antineoplastic drugs was a concern. Antiviral regimen enhancers (e.g., ritonavir, cobicistat) strongly inhibit cytochrome P4503A (CYP3A) affiliated with the cytochrome P450 (CYP) superfamily and enhance exposure to protease inhibitors, potentially enhancing exposure and toxicity of antineoplastic drugs, including antineoplastic agents metabolized by CYP3A, in addition to enhancing the efficacy of antiviral therapy. In addition, a retrospective analysis of HIV plus Hodgkin lymphoma showed that ritonavir-based HIV therapy and vincristine might cause irreversible neurological damage, in addition to non-nucleoside antiretroviral drugs that induce CYP3A, which could reduce anti-tumor exposure and toxicity. Therefore, the use of protease inhibitors and non-nucleoside antiretroviral drugs should be avoided as much as possible, and the choice of integrative medicine should be avoided. The anti-HIV regimen containing integrase inhibitors is more favored in malignant tumors, with little interaction with antitumor drugs and more rapid HIV-RNA inhibition, which is the optimal choice for antiviral therapy in patients with HIV-combined tumors. Considering that there are interactions between the HIV drugs and antitumor drugs, which affect the final therapeutic effect, the antiviral regimen was adjusted for the six patients receiving chemotherapy in this study, and the antiviral regimen was not used in the integrase inhibitor patients were due to economic difficulties and retreated to an antiviral regimen containing efavirenz or lopinavir/ritonavir.

The overall risk of cancer is higher in people living with HIV compared with the general population [6,7], and the risk increases with age [8]. This phenomenon is associated with chronic inflammation caused by the persistence of immunosuppressive conjugated viruses [9,10]. Aging due to increased life expectancy in the antiretroviral era leads to an increase in the incidence of non-AIDS-defined malignancies [11]. Non-AIDS-defined cancers account for approximately two-thirds of all cancers in people living with HIV, and their incidence is twice that of AIDS-defined cancers [7,9]. The Global Burden of Disease study reports a 45 and 26% increase in the incidence of non-Hodgkin lymphoma and leukemia, respectively, from 2006 to 2016 [10]. HIV-associated acute leukemia, although rare compared to the normal population, is more common in HIV patients than in the normal population [12,13]. From 2004 to 2020, we have reported one case of HIV-infected AML M4 [14], three cases of M2 [15,16], two cases of HIV-infected ALL L3 [16,17], and three cases of L2 [16,18], respectively. Of these nine patients, one was lost to follow-up, four had leukemia treatment failure, four died shortly after treatment, and the longest OS time among eight patients was 6 months. The diagnosis and treatment of HIV-infected acute leukemia is still in the exploratory stage and the occurrence of myeloid leukemia in AIDS patients may be related to various factors such as the effect of HIV on hematopoietic stem cells [19]. However, Guillemain et al. found that HIV RNA was not expressed in naïve cells in patients with HIV and acute monocytic leukemia. They hypothesized that HIV could not directly cause leukemia, and the appearance of leukemia may be related to virus-induced hematopoietic dysfunction and disruption of the bone marrow microenvironment [20]. Some scholars, such as Li, have suggested that cytokines such as tumor necrosis factor, interferon, and interleukins secreted by HIV-infected cells promote the malignant transformation of cells and may lead to myeloid leukemia [21]. In addition, it has been reported that treatment-related AML may occur in patients with AIDS-associated lymphoma after chemotherapy, and the cause analysis may be related to myelodysplasia and chemotherapeutic drug induction [22]. Wang et al. performed a statistical analysis of the age and gender of patients with non-HIV combined leukemia at the time of first visit in Harbin, Heilongjiang Province, in which AML accounted for 70.1%, ALL accounted for 29.9%, and the male-to-female ratio of leukemia was 1.2:1 [23]. The main symptoms of the patients in this study at the first visit were basically consistent with the common manifestations of patients with non-HIV infection and acute leukemia. The main symptoms were basically the same as those of non-HIV-infected patients with acute leukemia, but the male-to-female ratio, AML and ALL ratios were different, and the reasons for this need to be further explored. All four cases were later diagnosed with AIDS, suggesting that patients with AIDS may be diagnosed with hematologic manifestations and should be screened for HIV infection to avoid misdiagnosis and underdiagnosis. Testing for HIV infection is also recommended for anyone diagnosed with hematologic malignancies [24].

A multicenter study in France has found that there is no significant difference in the incidence of acute leukemia in HIV patients with the general population, but the onset of acute leukemia is earlier, with the average age of HIV patients with AML being 50 years [25]. The average age of onset of acute leukemia in this study was 43.9 years, which is earlier than the above conclusion, probably due to the small sample size, but suggests that early cancer screening in this group should be considered. Approximately 98% of APL translocations form the PML/RARa fusion gene, which has long and short types of transcripts. Short transcripts have poor efficacy, high early mortality, and easy recurrence, while long transcripts have a better prognosis [26], and PML/RARa gene mutations were present in all M3 cases in this study. Two patients had long fusion genes and a good prognosis after chemotherapy treatment, while one M3 patient failed chemotherapy and died. APL is a special type of AML characterized by coagulation abnormalities and a high cure rate. Although the prognosis for APL is currently good, early death remains a major challenge in the treatment of APL for the first time. Some studies have shown that the early morbidity and mortality of APL is 8.4%, and the median time from diagnosis to death is 7 (0–29) days. Age ≥ 50 years with WBC ≥ 10 × 10^9^/L at initial diagnosis is an independent risk factor for early mortality in patients with a first diagnosis of APL. Less than 87.1% of patients who died early had hemorrhage as the direct cause of death. Bleeding was the only cause of death for patients aged < 50 years. Patients aged ≥ 50 years old was the main cause of death [27]. Case 4 in this study, whose age and WBC count were consistent with the above results, died of intracerebral hemorrhage on the fourth day of admission. The other two M3 patients were treated in the hospital in a timely manner. Other two cases of M3 survived, with contrary results, suggesting that early identification of patients with possible leukemic comorbidities is extremely important and should be treated as soon as possible when suspicion of APL is high. If the patient’s physical condition is adequate, standard chemotherapy for APL in combination with ART is preferable [26]. Analysis of cause-of-death data in patients with a first diagnosis of hematologic malignancies in the United States has shown that tumors accounted for 56% of deaths, second oncology deaths accounted for 15%, non-oncological causes (e.g., cardiovascular events) accounted for 29%, and infectious causes accounted for 5% [27]. HIV with AML may progress more rapidly, and prompt treatment of the primary tumor is important. Aboulafia et al. reported 16 patients with HIV combined with AML karyotyping, 3 of whom had a normal karyotype, and the rest of the patients presented with abnormal karyotypes, including abnormal karyotypes, such as chromosome monosomy 7 and 16 inversion and T (17,19), and one patient with complex karyotype [28]. HIV with AML may progress more rapidly, and prompt treatment of the primary tumor is important. In the present study, four patients had karyotypic abnormalities that were more complex than those reported by Aboulafia et al. At least two chromosomes are abnormal, in many cases, as many as six chromosomes are abnormal, and subtetraploidy is also present. While in another case, case 10, where the primary tumor is NKT lymphoma, leukemia is a secondary tumor. The bone marrow karyotype shows the monosomy of chromosome 7. The long arm of one chromosome 5 is partially missing, and one chromosome 9 is inverted. The karyotype of the chromosome indicates a poor prognosis [29], due to the presence of nasal NKT lymphoma. Antiviral combined chemotherapy is still in a CR state after 70 months. To prevent rapid recurrence, hematopoietic stem cells or continuous chemotherapy is recommended [30]. Case 8 is ALL-L3, manifested as ectopic chromosome 8 and chromosome 14. Some studies have shown that chromosomal karyotype abnormalities in all patients are random. The common abnormal karyotypes are complex karyotype and *t*(9; 22) (Question 34; qll), the efficacy was poor, and 80 patients with ALL in this and with *t*(9;22) (q34;qll) karyotype, its efficacy are poor, this study 80 cases of ALL in *t*(8;14) (q24;q32) only 1 case (1.3%), accounting for a relatively small, and poor prognosis [31].

Certain anti-HIV drugs, such as protease inhibitors and NNRTIs, may enhance the exposure and toxicity of certain antineoplastic drugs by affecting the CYP3A isoenzyme in the cytochrome P450 (CYP) superfamily. Therefore, these drugs should be avoided as much as possible and integrase inhibitors with less interaction with antitumor drugs should be selected as the preferred antiviral treatment for patients with HIV infection and cancer. In the present study, six patients who received chemotherapy adjusted their antiviral regimens. Due to financial constraints, patients who do not take integrase inhibitors switch to antiviral regimens containing efavirenz or lopinavir/ritonavir. Discontinuation of HAART during leukemia treatment is not recommended because it may lead to further decline in immune function, opportunistic infections, and an increased risk of death. Sustained ART may lead to better cancer treatment tolerance, higher response rates, and improved survival.

In this study, four patients had lactate dehydrogenase exceeding the upper limit of normal. Higher lactate dehydrogenase levels are associated with greater tumor burden, faster disease progression, and higher rates of recurrence and mortality [32]. In patients with HIV combined cancer who require chemotherapy, discontinuation of HAART during chemotherapy is harmful in patients with HIV and cancer and may lead to poor outcomes despite increased toxicity and drug–drug interactions. Therefore, discontinuation of HAART is not recommended during leukemia treatment because of the potential increased risk of immunocompromise, opportunistic infections, and death. Continued ART may result in better cancer treatment tolerance, higher response rates, and improved survival. Therefore, it is best for HIV specialists to work with oncologists to develop a treatment plan. None of the patients in this study had ART discontinued during the anti-tumor therapy. HIV/AIDS with AML is rare, but hematologic abnormalities still need to be taken seriously by clinicians. The bone marrow karyotype analysis can all improve as soon as possible to confirm the diagnosis.

In this population, we have analyzed ART, standard systemic chemotherapy, infection prophylaxis, prompt use of strong antibiotics after chemotherapy, transfusion of component blood, as well as systemic supportive care. The analysis and treatment may improve the prognosis of patients with HIV and acute leukemia. Transplantation as part of standard treatment for hematologic malignancies is also an option for HIV-infected patients [33].
